# Recovery of Polyphenols from Agri-Food By-Products: The Olive Oil and Winery Industries Cases

**DOI:** 10.3390/foods11030362

**Published:** 2022-01-26

**Authors:** Paulina Tapia-Quirós, María Fernanda Montenegro-Landívar, Mònica Reig, Xanel Vecino, José Luis Cortina, Javier Saurina, Mercè Granados

**Affiliations:** 1 Department of Chemical Engineering and Analytical Chemistry, Universitat de Barcelona, Diagonal 645, 08028 Barcelona, Spain; paulina.tapia@upc.edu (P.T.-Q.); maria.fernanda.montenegro@upc.edu (M.F.M.-L.); xavi.saurina@ub.edu (J.S.); 2 Chemical Engineering Department, Escola d’Enginyeria de Barcelona Est (EEBE), Campus Diagonal-Besòs, Universitat Politècnica de Catalunya (UPC)-BarcelonaTECH, C/Eduard Maristany 10-14, 08930 Barcelona, Spain; monica.reig@upc.edu (M.R.); jose.luis.cortina@upc.edu (J.L.C.); 3 Barcelona Research Center for Multiscale Science and Engineering, Chemical Engineering Department, Escola d’Enginyeria de Barcelona Est (EEBE), Campus Diagonal-Besòs, 08930 Barcelona, Spain; 4 Chemical Engineering Department, Research Center in Technologies, Energy and Industrial Processes—CINTECX, Campus As Lagoas-Marcosende, University of Vigo, 36310 Vigo, Spain; 5 Water Technology Center—CETAQUA, Carretera d’Esplugues, 75, 08940 Cornellà de Llobregat, Spain

**Keywords:** olive mill wastes, winery wastes, phenolic compounds, circular economy, resource recovery, extraction process, membrane technology, resins

## Abstract

The production of olive oil and wine are two of the main agri-food economic activities in Southern Europe. They generate large amounts of solid and liquid wastes (e.g., olive pomace, olive mill wastewater, grape pomace, grape stems, wine lees, and wine processing wastewater) that represent a major environmental problem. Consequently, the management of these residues has become a big challenge for these industries, since they are harmful to the environment but rich in bioactive compounds, such as polyphenols. In recent years, the recovery of phenolic compounds has been proposed as a smart strategy for the valorization of these by-products, from a circular economy perspective. This review aims to provide a comprehensive description of the state of the art of techniques available for the analysis, extraction, and purification of polyphenols from the olive mill and winery residues. Thus, the integration and implementation of these techniques could provide a sustainable solution to the olive oil and winery sectors.

## 1. Introduction

Agri-food industries bring about a large amount of waste. It is estimated that around 90 million tons of food waste are generated in the European Union each year, which constitutes a serious problem for the environment [1]. However, it is well known that agri-food waste and by-products can be sources of valuable compounds, such as polyphenols, with relevant functional biological activities, such as antioxidant, anticancer, antihypertensive, or anti-cholesterol [2,3,4].

**Figure 1:**
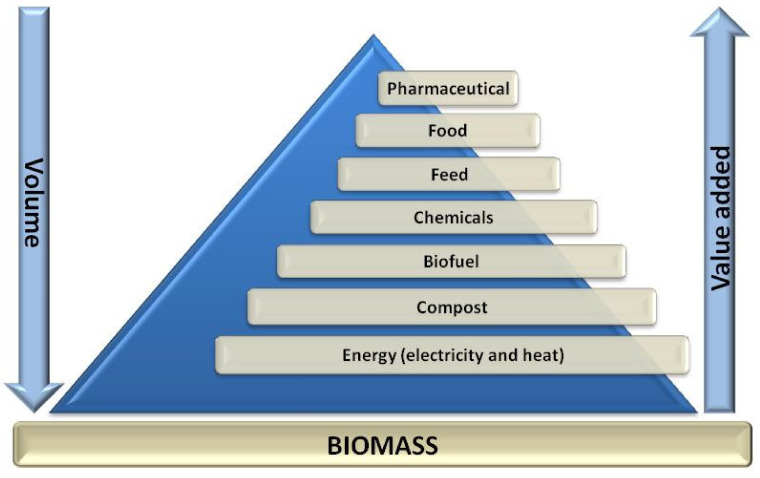
Pyramid of biomass value (adapted from [5]).

Bioeconomy covers the production of renewable biological resources and the conversion of these resources and wastes into value-added products, maintaining the value of products and materials for as long as possible while minimizing the use of resources and the generation of wastes [5]. Biomass volume or mass and its added value are inversely related. Thus, in the bottom part of the pyramid of biomass (see Figure 1) the most common application can be found, i.e., the use of biomass as an energy source, which has the lowest priority since it provides the lowest value (15–40 €/ton). Conversely, in the top part, the biomass used in pharmacy and cosmetics could result in products that have associated the highest values (e.g., 10,000–20,000 €/kg). Food and feed find the second and third place respectively, and chemicals are in the fourth position [5].

Phenolic compounds are products of high added value, with potential applications in the pharmaceutical, cosmetic, and food industries, and their recovery from waste can be highly interesting [6]. Studies indicate that consuming polyphenols reduces the risk of degenerative diseases, by inhibiting the oxidation of low-density lipoproteins [7]. Polyphenols may also offer protection against the development of cancer, cardiovascular diseases, diabetes, or osteoporosis [6]. The antioxidant capacity of the polyphenols correlates with their chemical structure; in general, they mainly prevent the formation of free radicals connected involved in the processes of autoxidation, by donating hydrogen atoms or electrons [4].

Phenolic acids and polyphenols are secondary metabolites of plants, generally related to the defense against ultraviolet radiation, pathogens, and environmental stress [7,8]. Polyphenols are characterized by the presence of more than one phenol group per molecule, as well as other possible functional groups. However, the term polyphenols are quite often used in a broader sense, encompassing phenolic acids.

**Figure 2:**
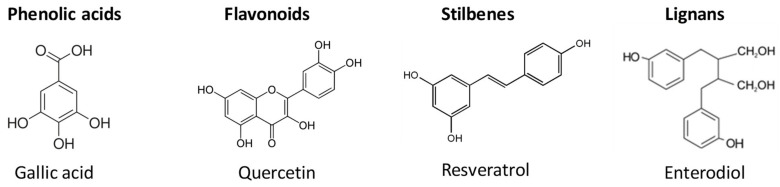
Chemical structures of phenolic acids and polyphenols representative of the main families.

Most of the phenolic metabolites may be classified into four large groups: phenolic acids, flavonoids, stilbenes, and lignans, as shown in Figure 2 [4].

Phenolic acids have a carboxylic acid functional group and can be divided into hydroxybenzoic acids and hydroxycinnamic acids, based on their C1-C6 and C3-C6 structures, respectively. The latter group includes compounds such as caffeic, ferulic, *p*-coumaric, chlorogenic, or synaptic acids, while hydroxybenzoic acids include, among others, *p*-hydroxybenzoic, protocatechuic, syringic, vanillic, and gallic acids, and related tannins hydrolyzable tannins [8].

Flavonoids consist of two aromatic rings that are linked by three carbon atoms that form an oxygenated heterocyclic ring. Depending on the type of heterocyclic involved, they can be classified into different subclasses: flavonols, flavones, isoflavones, flavanones, anthocyanidins, and flavanols [4].

Stilbenes are a group of compounds derived from phenylpropanoids, characterized by the main chain of 1,2-diphenylethylene (C6-C2-C6). They are present in the human diet in low quantities and the main representative is resveratrol, detected in more than 70 species of plants, including grapes, berries, and peanuts [4].

Lignans are produced by oxidative dimerization of two units of phenylpropane. These compounds are considered phytoestrogens, in addition to their anti-inflammatory, antioxidant or antitumor properties, or their beneficial effects in preventing cardiovascular diseases. Sesame and flaxseed are rich sources of lignans in the human diet [9].

Apart from these four main families, other minor groups of compounds related to polyphenols include the secoiridoids, which are bicyclic monoterpenes (C10) derived biosynthetically from geraniol. Olives contain secoiridoid compounds, with oleuropein being the majority [10]. Phenethyl alcohols, like tyrosol and hydroxytyrosol, are also found in olives [11].

As polyphenols are plant metabolites, they are present in agri-food waste, which can be revalued when the recovery of these bioactive compounds is considered. This review is focused on the recovery of polyphenols from waste related to the production of olive oil and wine, two of the main sectors of the agri-food economy in southern Europe.

## 2. Polyphenols from Olive Oil Production Wastes: Source and Applications

Olive oil is defined as the oil obtained from the olive, which comes from the *Olea europaea* L. tree. It is an edible oil of high consumption, with volumes that have increased steadily since 2005. In the last decade, the production of olive oil has increased by about 40% worldwide. The main olive oil producers are Spain, Italy, Greece, and Portugal. The growing popularity of olive oil is predominantly attributed to its content of oleic acid and phenolic compounds, being considered a valuable source of antioxidants in the human diet. Nevertheless, during its production, a considerable amount of phenolic compounds remains in olive oil by-products and waste [12,13].

**Figure 3:**
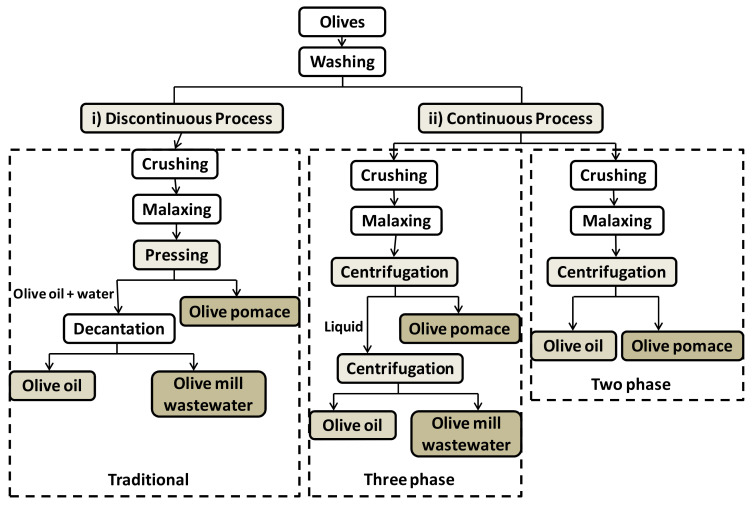
Olive oil processing schemes: i) traditional as discontinuous process and ii) continuous processes by three phases or two phases (adapted from [12]). Reproduced with permission from S. Dermeche, et al. Process Biochemistry; published by Elsevier, 2013.

The olive oil industry generates large amounts of solid and liquid wastes, olive pomace and olive mill wastewater being the main ones. All of them have a high load of lipids, organic acids, and phenolic compounds. Figure 3 shows the main olive oil processing schemes and the main streams of interest as a source of polyphenols.

The chemical composition of waste depends on several factors, such as cultivation, origin, maturation of the olive, climatic conditions, and olive oil extraction procedure. Several studies have shown the negative effects of waste on microbial populations of the soil, aquatic ecosystems, and the air through the emissions of phenol and sulfur dioxide, so that waste constitutes a major environmental problem in the main producing countries of olive oil from the Mediterranean region [1,12].

One of the main approaches for the valorization of these residues is the extraction of valuable phytochemical compounds, such as polyphenols, with interesting properties for the pharmaceutical, cosmetic, and food industries [12]. The main phenolic compounds identified in residues of olive oil production are shown in Table 1.

As can be seen in Figure 3, olive pomace and olive mill wastewater are produced as by-products, regardless of the processing scheme followed.

Olive pomace is a semi-solid waste, composed of olive skin, pulp, and bone. The main components are polysaccharides, proteins, fatty acids, pigments, and polyphenols, and thus it is considered a source of compounds with antioxidant activity [17]. Olive pomace is rich in hydroxytyrosol but also has a significant content in oleuropein, tyrosol, caffeic acid, *p*-coumaric acid, vanillic acid, verbascoside, elenolic acid, catechol, and rutin [18]. It is estimated that olive pomace production is higher than 2.8 million tons/year worldwide [18,19].

Olive mill wastewater is the liquid residue composed by the olive washing waters, olive pulp water, water added to olive paste in the centrifugation step of the three-phase scheme, and water from the washing of extraction plants [20]. The production of olive mill wastewater is estimated between 7 and more than 30 million m^3^ per year worldwide [12]. This waste is constituted by sugars, polyalcohols, lipids, pectins, polyphenols, etc. The main phenolic compound is hydroxytyrosol but also is rich in tyrosol, oleuropein, caffeic acid, vanillic acid, gallic acid, luteolin, and verbacoside, among others [13,21].

### Applications of Polyphenols from Olive Oil Industrial Wastes

Research into the application of olive by-products and/or their bioactive compounds is growing [22], aimed to improve the nutritional profile of food products, to improve food properties, to obtain innovative natural additives for cosmetics, etc.

As stated previously, hydroxytyrosol is widely present in olive oil by-products, and its antioxidant and antimicrobial properties have been widely demonstrated [1,5,12,13,18]. In this sense, Ruiz-Moreno et al. [23] tested the extract from olive waste, rich in hydroxytyrosol, as a potential substitute for sulfur dioxide in winemaking. After verifying that the most important odorants present in the extract were also present in wines, they observed that the extract showed antimicrobial activity against *Hanseniaspora uvarum*, *Candida stellata*, *Lactobacillus plantarum*, *Pediococcus damnosus*, *Acetobacter aceti*, *Oenococcus oeni*, *Dekkera bruxellensis*, and *Botryotinia fuckeliana* microorganisms.

On the other hand, the food industry is interested in adding natural antioxidants to packaging materials to preserve food products and extend their shelf-life, while maintaining the organoleptic properties. In this sense, the food packaging sector is developing active materials based, for instance, on by-products of agribusiness, such as lignocellulosic fibers from olive pomace [18]. Instead, Luzi et al. [24] developed ternary films for food active packaging, using polyvinyl alcohol as a polymeric matrix, nanostructured starch as reinforcement phase, and hydroxytyrosol as an antioxidant agent. Experimental data showed a prolonged release of hydroxytyrosol from ternary films with strong antioxidant activity, showing favorable results for active food packaging. Cejudo et al. [25] used an olive leaf extract as the active component in polyethylene terephthalate/polypropylene films, oleuropein and luteolin-7-glucoside being the main phenolic antioxidants. The effectiveness of the film against lipid oxidation was evaluated during the storage of sunflower seeds, obtaining, as a result, the delaying of lipid oxidation.

As a matter of example, focusing on the cosmetic industry, Galanakis et al. [26] studied the application of different concentrations of phenolic compounds, such as hydroxytyrosol, tyrosol, or oleuropein, recovered from olive mill wastewater as boosters of UV filters. Absorption of synthetic UV filters increased as a function of olive phenols concentration, while the entrapment of olive phenols in silica particles, improved their water resistance.

## 3. Polyphenols from Wine Production Wastes: Source and Applications

Wine production is one important agricultural activity, *Vitis vinifera* being the most cultivated species for wine production. Wine production regions are mainly located in Europe (Italy, Spain, France, Germany, and Portugal) and America (USA, Argentina, and Chile), but also in Australia and South Africa [7].

During vinification, only 30–40% of the phenolic compounds are extracted [8], and the process generates different types of waste and by-products such as grape pomace, grape stems, wine lees, and wastewater. One ton of grapes generates approximately 0.13 t of pomace, 0.03 t of stems, 0.06 t of lees, and 1.65 m^3^ of wastewater [7,27].

Although the residues of wine production are mainly produced in the harvest period, they are distributed throughout the year causing environmental problems if they are not properly disposed of. These residues, which have commonly been considered a problem, are rich in bioactive compounds with potential uses as food additives, nutraceuticals, and/or cosmeceuticals [7]. The main phenolic compounds identified in wine production waste are shown in Table 2.

**Figure 4:**
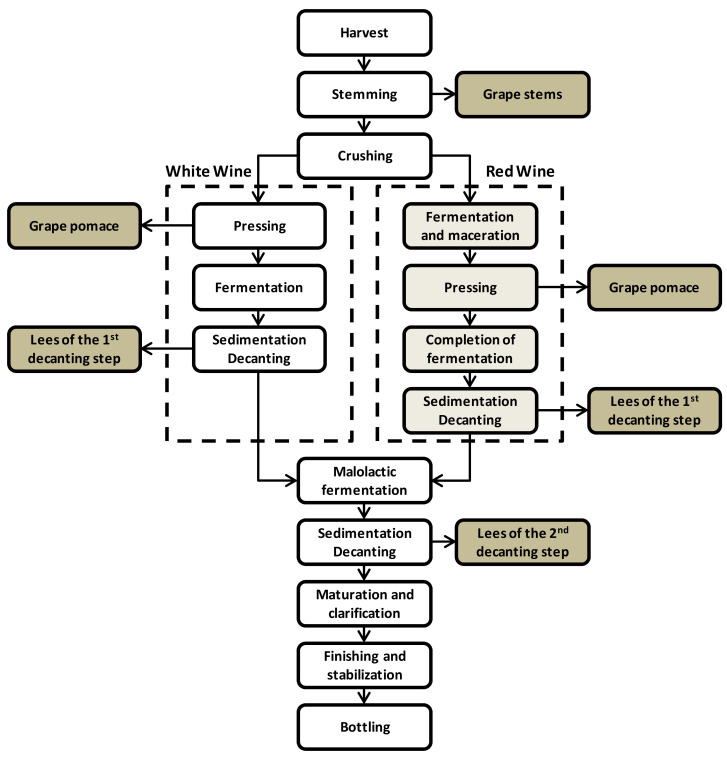
Wine processing schemes (adapted from [33]). Reproduced with permission from R. Devesa-Rey, et al. Waste Management; published by Elsevier, 2011.

At present, the valorization of winery by-products is mainly represented by their use as soil fertilizers, production of biomass, and animal feeding. Nevertheless, some compounds present in winery by-products are phytotoxic and show antimicrobial effects during composting, which affect their use for this purpose. Regarding its use in animal feed, certain components, such as condensed tannins, negatively affect the digestibility of animals [31,32]. Therefore, its valorization as a source of bioactive compounds for application in the pharmaceutical, cosmetic, and food industries is a smart alternative [31]. The main types of wastes generated in the winery industry are grape pomace, grape steams, wine lees, and wine processing wastewater. The main wine processing schemes and the main streams of interest as a source of polyphenols are described in Figure 4.

As depicted in Figure 4, the main waste streams of interest for potential polyphenol recovery are grape pomace, clusters or grape stems, wine lees, and wine processing wastewater.

Grape pomace is the residue that originated during the pressing of the grapes to produce must. It is constituted by the skins and seeds of the grapes. During the vinification process, a large amount of grape pomace is generated. Nowadays, 9 million tons of this organic waste are produced per year in the world, which constitutes, on average, 20% of the total grapes used for wine production [31]. This residue constitutes an important source of phenolic compounds, mainly anthocyanins, flavonols, flavonoids, phenolic acids, and stilbenes [8]. Grape pomace is used for the production of citric acid, methanol, ethanol, and xanthan through fermentation, and it is also employed for the production of energy [7,31]. Several studies have reported a high antioxidant activity of this by-product, suggesting that grape pomace is an interesting source of natural antioxidants [31].

Clusters or grape stems constitute a residue of the wine industry that is used as a source of astringent compounds, represented mainly by proanthocyanidins. This residue is eliminated before the vinification step to prevent an excessive wine astringency or a negative effect on organoleptic characteristics [31].

Wine lees are generated during the fermentation and maturation processes of the wine. They are composed of solid and liquid fractions. The solid fraction contains the deposits formed at the bottom of the tanks, which mainly consist of yeasts and bacteria, carbohydrates, phenolic compounds, lignin, proteins, metals, inorganic salts, salts of organic acids (e.g., tartrates), and grape residues. On the other hand, the liquid phase mainly consists of the spent fermentation broth; therefore, it is rich in ethanol and organic acids. Besides, lactic acid from malolactic fermentation and acetic acid may also be present in significant amounts [27]. Finally, the vinasses are the residual liquid fraction of the wine lees distillation process, which is carried out to recover ethanol and produce distilled beverages [27].

Wine processing wastewater is a residue generated during different stages of wine production: fermentation, storage, and maturation (washing tanks and containers), clarification (wastewater generated by filtration), decanting, and bottling (spills and cleaning of containers and bottles) [34]. Wine production generates large amounts of wastewater; between 0.5–14 L per liter of wine produced. They are mainly acidic, phytotoxic with a high biological oxygen demand (BOD) and a relatively high concentration of phenols [34,35]. The mean values of chemical oxygen demand (COD) and BOD are 11.886 mg L^−1^ and 6.570 mg L^−1^, respectively [34,36].

### Applications of Polyphenols from the Wine Industry

Winery waste extracts and grape by-products can be applied to foods, pharmaceuticals, or cosmetics, either in the form of liquid, concentrated, or powder extracts [8,31,37,38,39].

The polyphenolic content of grapes has attracted the interest of the pharmaceutical, cosmetic, and food industries as a profitable source of natural antioxidants [31]. Currently, there are cosmetics or food supplement products marketed with grape polyphenols [8]. Matos et al. [39] demonstrated that red wine lees extracts exhibited the highest antioxidant capacity and inhibitory effect to enzymes enrolled in the skin aging process (elastase, collagenase, and tyrosinase), and the protection of human skin cells (keratinocytes and fibroblasts) against oxidative stress.

In the health field, there are studies about the effect of polyphenols from natural sources in the prevention and treatment of cancer [8,40,41,42]. For instance, Hamza et al. [40] provided evidence that grape seed extract has an anticancer effect in liver cancer by the inhibition of cell proliferation, induction of apoptosis, modulation of oxidative damage, and suppression of inflammatory response. Additionally, Li et al. [41] found that resveratrol, a stilbene present in grapes, inhibits the proliferation of human cervical carcinoma cells and elevates apoptosis. Therefore, resveratrol may be a promising inhibitor of human cervical cancer.

Likewise, grape pomace extracts have been successfully incorporated into edible chitosan films, providing antioxidant properties and prolonging shelf life in foods, as shown by Alves et al. [37]. They prepared chitosan films with grape seed extract and carvacrol microcapsules and tested their physicochemical properties and their effect on physico-chemical and microbiological parameters in refrigerated salmon. It was observed that the chitosan films increased the shelf-life of refrigerated salmon from 4–7 days of storage due to the antimicrobial effect of the natural agents. Additionally, Fabra et al. [43] developed active edible films with antiviral activity by adding grape seed extract as an active agent in alginate-lipid films. The films exhibited significant antiviral activity against murine norovirus and hepatitis A virus. Furthermore, the addition of grape seed extract exhibited strong antioxidant activity to the active films and can improve the food quality and safety.

The recovery of polyphenols from waste, whatever their type, generally requires the implementation of extraction and purification processes, in addition to having an analytical methodology to control the processes and the quality of the products.

## 4. Polyphenol Analysis: From Quantification to Antioxidant Capacity

The determination of polyphenols can be approached from two main perspectives: by liquid chromatography, which provides the polyphenolic profile, but also allows the quantification of target individual polyphenols, or by spectrophotometric or electrochemical assays, which provide an estimation of the total polyphenol content or the antioxidant capacity.

### 4.1. Chromatographic Techniques

High-performance liquid chromatography (HPLC) or ultra-high-performance liquid chromatography (UHPLC) is frequently used to analyze polyphenolic compounds. There are many studies of identification and quantification of polyphenols in olive oil [44,45,46,47,48,49,50,51,52,53,54] and wine by-products [30,55,56,57,58,59,60,61,62] by chromatographic techniques.

**Figure 5:**
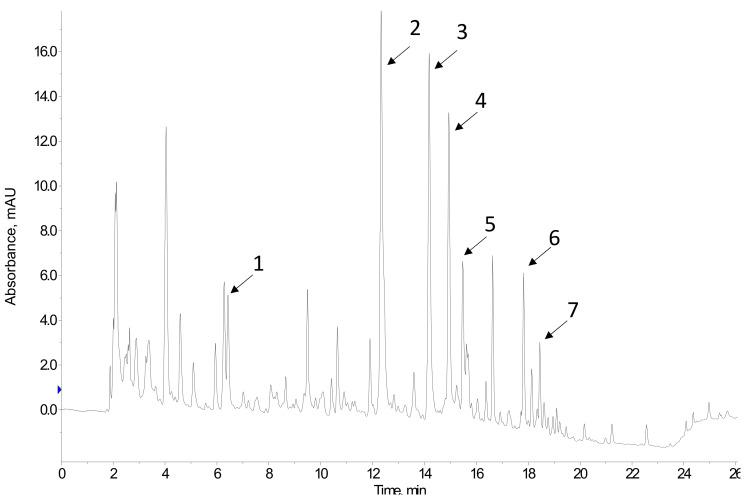
HPLC-UV chromatogram at 280 nm of a wine lees extract. Peak assignment: 1 Gallic acid; 2 catechin; 3 cis-coutaric acid; 4 trans-coutaric acid; 5 caffeic acid; 6 p-coumaric acid; 7 astilbin.

Commonly, separation is performed in the reverse phase mode, using C18 columns, and with gradient elution using mobile phases based on methanol:water or acetonitrile:water containing formic acid [63]. Different gradient programs are proposed, depending on the polyphenolic profile of the samples. The most frequently used detectors are ultraviolet (UV)-diode array (DAD) and mass spectrometry (MS) detectors, but fluorescence and electrochemical detectors are also applied. Figure 5 shows the HPLC-UV chromatogram of a wine lees extract.

It is well known that MS allows the confirmation of the identity of the polyphenols. In this sense, high-resolution mass spectrometry (HRMS) is especially suited when dealing with complex samples, such as extracts from vegetal origin products. Thus, the HRMS spectrum and the retention time are used for confirmatory analysis [63,64]. It must be said that low-resolution MS/MS systems are also applicable. Both low- and high-resolution MS are suited for quantitative analysis, providing excellent selectivity as well as sensitivity, but it must be kept in mind that, to obtain reliable quantitative results, calibration should account for potential matrix effects, which are quite common in liquid chromatography LC-MS.

UV detection is simpler than MS detection, but the UV spectrum provides useful information which, combined with retention time, allows for tentative identification of compounds. As a matter of example, Fernandez et al. [54] identified luteolin, hydroxytyrosol, trans-ferulic acid, rutin hydrate, tyrosol, apigenin, and caffeic acid in olive pomace samples by HPLC-DAD-UV. Romero et al. [48], also using HPLC-DAD-UV, reported the presence of hydroxytyrosol-4-glucoside, hydroxytyrosol, dialdehydic form of decarboxymethyl elenolic acid linked to hydroxytyrosol (HyEDA), verbascoside, tyrosol, and salidroside in olive pomace samples. Moreover, Jurčević et al. [60] identified quercetin, ellagic acid, gallic acid, caffeic acid, p-coumaric acid, chlorogenic acid, and kaempferol in wine lees samples by HPLC-UV. If the chromatographic peaks are well resolved, UV detection can be used for quantitative analysis of target compounds [63]. HPLC-UV quantitative analysis is simpler than in the case of MS since there are no matrix effects.

A different approach is related to the analysis of total polyphenol content (TPC). It is also based on the use of UV detection. The chromatogram is acquired at a wavelength where polyphenols absorb (e.g., 280 nm or 320 nm), and the total area of the peaks eluting in the time window, where elution of polyphenols occurs, is calculated. This area is related to TPC, which is estimated using a calibration curve obtained with a standard (e.g., gallic acid). The TPC is expressed in terms of the equivalent concentration of the standard used in calibration [65].

### 4.2. Spectrophotometric Methods

The most widely applied method to determine the TPC in vegetal products is Folin-Ciocalteu (FC) [66]. There are also spectrophotometric assays to determine the antioxidant capacity or activity, which in samples of agri-food waste is largely due to the presence of phenolic compounds. The assays can be classified according to the mechanism of the chemical reaction involved in the method: hydrogen atom transfer (HAT) and single electron transfer (SET). In HAT assays the measure is related to the capability of antioxidants (e.g., phenolics) to scavenge free radicals by donating a hydrogen atom. SET assays measure the ability of antioxidants to transfer electrons to oxidized forms (metal ions, radicals, etc.). Some assays involve both HAT and SET mechanisms.

#### 4.2.1. Folin-Ciocalteu Method

The FC method is a SET assay based on the reaction of phenolic compounds with the FC reagent in alkaline conditions. This reagent is a mixture of phosphomolybdate and phosphotungstate, yellow in color, which, when reduced by the phenolic groups, gives rise to an intense blue complex, with a broad absorption band in the 600–800 nm range. Diverse wavelengths in this range have been reported for the spectrophotometric analysis. The measured absorbance is proportional to the polyphenol content in the sample, which is usually quantified based on a calibration curve of gallic acid, and the results are expressed in terms of gallic acid equivalent (GAE) concentration. The assay is not specific for polyphenols, and other reducing compounds (e.g., carotenoids, vitamin E, vitamin C, reducing sugars, certain amino acids) interfere [13,67]. However, it is assumed that the reaction of the FC assay with agri-food products is mostly due to polyphenols, and therefore the FC index is a good estimation of polyphenols content for such samples, as evidenced by the correlations reported for FC results and those obtained by other techniques such as LC-UV [66,68].

There are different studies on the analysis of TPC by the FC method in residues of olive oil production. For instance, Elkacmi et al. [69] determined the TPC in olive mill wastewater, obtaining values of 2.1–6.43 g GAE L^−1^. For olive pomace, Alu’datt et al. [45] and Razek et al. [47] reported TPC values of 0.06–4.37 mg GAE g^−1^ dry weight (dw) and 69.66 mg GAE g^−1^ dw, respectively. There are also many studies for TPC determination in residues of wine production by the FC method, which is dependent on the extraction procedure used. As a matter of example, some values reported for TPC are 3.22 mg GAE g^−1^ dw in grape skin [55], 42.10–188.21 mg GAE g^−1^ wet weight (ww) [70], 50.96–219.23 mg GAE kg^−1^ dw [57], and 196.2 mg GAE g^−1^ dw [58] in grape pomaces, 63.46 mg GAE g^−1^ ww in grape steams [70]_,_ 188.21 mg GAE g^−1^ ww in grape seeds [70], and 26.1 g GAE g^−1^ dw [71], 9.84–23.17 mg GAE g^−1^ dw [60] in wine lees_._

#### 4.2.2. 2,2-Diphenyl-1-picrylhydrazyl (DPPH) Assay

The DPPH assay is a mixed-mode HAT/SET, used to determine the antioxidant capacity. DPPH is a free radical that has a deep purple color; it is one of the few stable organic radicals. The assay is based on the reaction of the DPPH radical with an antioxidant, e.g., phenolic compounds, which generates a reduced molecular form of the radical, and results in the loss of the purple color. Thus, the decrease in absorbance at 515 nm depends on the concentration of the antioxidant. Trolox is used as standard in calibration and the results of antioxidant capacity are expressed in terms of Trolox equivalent (TEAC) concentration [72,73,74,75]. Tournour et al. [76] evaluated the antioxidant capacity by DPPH in ethanol/water extracts and aqueous suspensions of grape pomaces from four different Portuguese grape varieties. The highest DPPH values were found in Touriga Nacional grape cultivar with 1.09 ± 0.13 and 1.12 ± 0.04 mmol TEAC g^−1^ dry residue, in ethanol/water extracts and aqueous suspensions, respectively.

#### 4.2.3. 2,2′-Azino-bis(3-ethylbenzothiazoline-6-Sulfonic Acid) Assay

In this method, the 2,2′-azino-bis(3-ethylbenzothiazoline-6sulfonic) acid (ABTS) is used. Antioxidants reduce the cation radical (ABTS^·+^), throughout mixed-mode HAT/SET mechanisms, and a change of color occurs from a bluish-green solution to a decolorized one. The absorbance diminution at 743 nm depends on the concentration of antioxidants in the sample. Trolox is commonly used as a standard in calibration, and the ABTS results are expressed in terms of Trolox equivalent concentration [72,73,74,75,77]. Poveda et al. [70] applied the ABTS assay to the analysis of winery by-products, such as grape seeds, pomace, and stems. The highest antioxidant capacity values were found in grape seeds extracts (0.061–0.065 mmol Trolox g^−1^). Similarly, Abdel-Razek et al. [47] applied the ABTS assay to evaluate the antioxidant activity of olive oil production by-products (olive pomace, olive leaves, and pomace olive oil). The highest antioxidant capacity was observed in olive pomace samples.

#### 4.2.4. Ferric Reducing Antioxidant Power (FRAP)

FRAP assay is a colorimetric method that measures the antioxidant capacity of a sample based on a redox reaction. The reaction involves a SET mechanism and consists of the reduction of ferric tripyridyl triazine complex to ferrous form at low pH (3.6), which has an intense blue color and can be monitored by measuring the change in absorption at 593 nm. The increase of absorption is related to the concentration of antioxidants in the sample. Trolox is used as standard and results are expressed in Trolox equivalent concentration [72,75,77]. Jurčević et al. [60] evaluated the potential of wine lees polyphenols as novel functional bioactive compounds in the protection against oxidative stress and hyperlipidemia. The antioxidant capacity of wine lees determined by FRAP was 45.7 ± 1.05 mM of Trolox equivalents per 100 g, respectively.

#### 4.2.5. Oxygen Radical Absorbance Capacity (ORAC)

The ORAC method is a HAT assay used to determine antioxidant activity. It is based on the quenching of the fluorescence of fluorescein by peroxyl radicals. In the presence of antioxidants, which react with peroxyl radical, the decrease in the fluorescence signal is slowed down. The fluorescence decay over time is recorded, and the area under the curve is related to the antioxidant capacity. Trolox is habitually used as the standard in calibration, and results are reported as Trolox equivalent concentration [72,73,77]. Antoniolli et al. [58] determined the antioxidant capacity by ORAC of grape pomace extracts of cv. Malbec, and reported 2756 µmol TEAC g^−1^. Pasten et al. [78] analyzed the antioxidant activity of Arbequina olive residues. The antioxidant capacity of fresh samples was 66.81, 101.75, and 283.5 µmol TEAC g^−1^ dw, for DPPH, FRAP, and ORAC, respectively.

**Figure 6:**
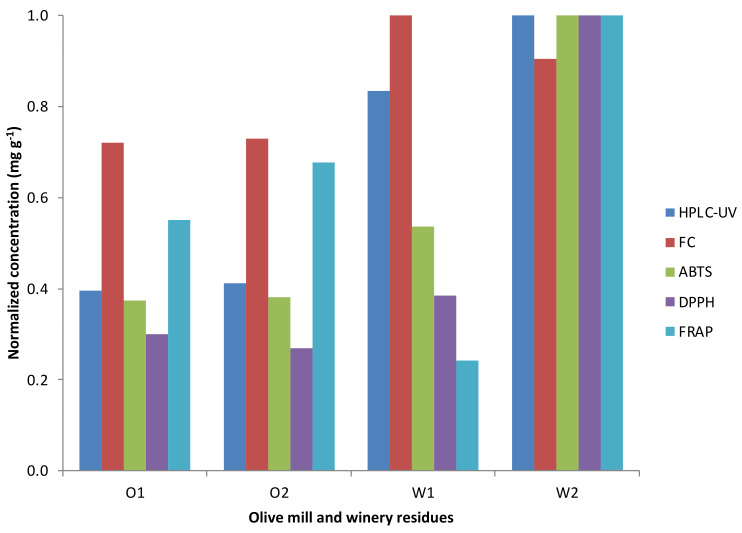
Normalized results of the spectrophotometric assays and HPLC-UV technique from the olive mill (O1 and O2) and winery residues (W1 and W2).

It is not evident what is the equivalence of the results provided by the different assays. It is not just a question of the units used to express the results; quite often, there is not a clear correlation between the results from different assays. However, as a general trend, when comparing results from different samples measured by several assays, samples having high results in one assay usually show high values in the others (See Figure 6).

Finally, it should be mentioned that, although less applied than spectrophotometric assays, electrochemical assays can also be used to determine the antioxidant capacity of samples, with cyclic voltammetry, differential pulse voltammetry, and square wave voltammetry being the most widely used techniques [79]. In this sense, electrochemical detection combined with molecularly imprinted polymers (MIPs) is an innovative approach for the quick detection of antioxidants [80].

## 5. From Agri-Food Wastes to Polyphenols: Processing Techniques

**Figure 7:**
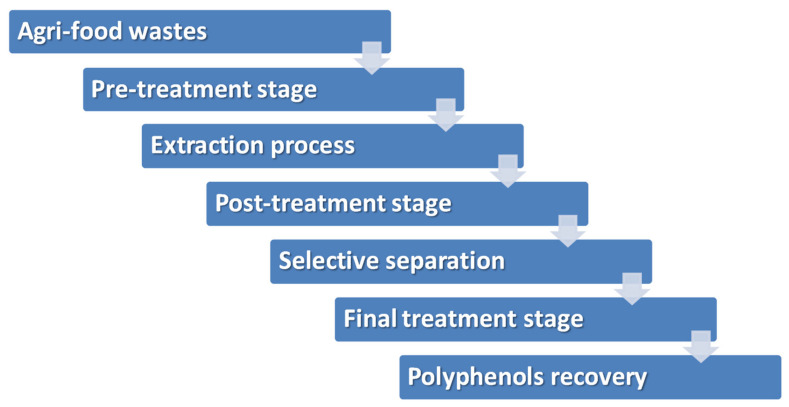
Recovery of polyphenols from agri-food wastes.

Recovery of polyphenols from agri-food wastes should be considered as a multi-stage process where the more relevant stages are: i) extraction of the polyphenols from the solid matrix when the waste is a solid or pulp type; ii) post-treatment of the generated extracts by membrane technologies; iii) selective separation of mixtures of polyphenols or individual components; and iv) final treatment stages of lyophilization to produce powder forms (Figure 7). The most critical are the first three stages; thus, the state of the art of such stages are described following.

### 5.1. Extraction Processes

Several techniques have been used to recover polyphenols from olive oil and wine by-products. Conventional extraction with solvents, also called solid-liquid extraction (SLE) or maceration, is the most applied technique from an industrial point of view, to extract bioactive compounds from matrices of vegetable origin [7,81]. Many solvents have been studied for polyphenols extraction, but the preferred systems for food, pharmaceutical, or cosmetic applications are those based on water and ethanol [82].

New non-conventional techniques appeared due to the growing need for the use of more efficient recovery procedures. These new methods can reduce extraction time, process temperature, and solvent consumption, thus contributing to a higher extraction efficiency and lower consumption of energy. Some of the most relevant technologies are: ultrasound-assisted extraction (UAE), microwave-assisted extraction (MAE), supercritical fluid extraction (SFE), pressurized liquid extraction (PLE), enzyme assisted extraction (EAE), ohmic heating (OH), and pulsed electrical fields (PEF) [1,82,83,84,85,86].

Ultrasound-assisted extraction (UAE). UAE consists of the use of high-frequency waves (≥ 2 MHz) that produce negative pressure, change the physicochemical properties of the medium, and allow the formation of cavitation bubbles. This last phenomenon is responsible for the rupture of the cell membranes in the samples, promoting contact between the solvent and the matrix. The use of this technique is increasing because of cavitation, which improves the transfer of heat and mass through the rupture of cell walls. UAE can be done using an ultrasonic bath, or more efficiently with an ultrasonic probe [7].

Microwave-assisted extraction (MAE). MAE is an extraction method that is based on the direct impact of microwave radiation on polar molecules [7]. The microwave energy is used to heat the polar solvents in contact with solid samples and to extract compounds of interest from the sample to the solvent, increasing the internal pressure inside the cell. This facilitates the rupture of the cell wall and the release of active compounds to the solvent [1].

Supercritical fluid extraction (SFE). The extraction with supercritical fluids is a technology that replaces the use of organic solvents with carbon dioxide in a supercritical state. The increase of the pressure and the temperature of the liquid/gas, above the critical point, generate fluids in the supercritical state. CO_2_ is the most widely used supercritical fluid due to its availability, non-toxicity, and flammability, low cost compared to liquid organic solvents, and easiness to handle critical temperature and pressure (31.1 °C and 7.38 MPa). When compared to liquid organic solvents, supercritical CO_2_ has a relatively low viscosity, high molecular diffusivity, and low surface tension of the system. All these properties lead to the improvement of mass transfer. Due to the non-polar character of CO_2_, it is especially suited for the extraction of non-polar compounds. The addition of a co-solvent, such as ethanol, methanol, acetone, increases the polarity of CO_2_ and allows the extraction of polar compounds [1,7].

Pressurized liquid extraction (PLE). PLE is based on the use of the extraction solvent at high temperatures and pressure. Working at high pressure allows maintaining the solvent in a liquid state at temperatures above its boiling point. This technique can be used in three modes: dynamic, static, and dynamic-static. In dynamic mode, the solvent is constantly driven through the cell during extraction; in the static mode, the solvent is only pumped between the extraction cycles; while the dynamic-static mode represents a combination of both [13].

Enzyme assisted extraction (EAE). Some enzymes, such as cellulases, hemicellulases, pectinases, or amylases, can break down or weaken cell walls, releasing cytoplasmic content (e.g., phenolic compounds) into the extraction solvent, and thus improving extraction recoveries. Factors such as enzyme type and concentration, pH, or temperature have an influence on extraction recovery. EAE can also be combined with other extraction techniques such as UAE, MAE, PLE, or SFE [87].

Ohmic heating (OH). In OH the material is in direct contact with the electrodes and an alternating electrical current, with a frequency between 50 and 100 kHz, passes throughout the material therefore rapid and uniform heating occurs. OH has found several applications in food technology, and it also has been applied to the extraction of phenolic compounds from agri-food wastes [85].

Pulsed electrical fields (PEF). PEF uses high voltage, between 10–40 kV, for an extremely short time, in the range of a few µs, producing a strong electric field. It is considered a non-thermal technology because in PEF the temperature rise is usually low—a few °C. PEF produces electroporation of the cell membrane, and consequently the permeation throughout the membrane increases, and thus extraction improves. Parameters such as the strength of the electric field, the amplitude of the pulses, the duration of the pulses, etc., have an effect on the efficiency of the extraction [88].

Examples of the application of the different techniques and solvents to the extraction of polyphenols from the olive mill and winery wastes are presented in Table 3.

For instance, Xie et al. [97] compared UAE, MAE, and SLE for the extraction of 3-hydroxytyrosol, maslinic acid, and oleanolic acid from olive pomace. As a result, UAE yielded the maximum extraction of 3-hydroxytyrosol (55.1 mg g^−1^), maslinic acid (381.2 mg g^−1^), and oleanolic acid (29.8 mg g^−1^), followed by MAE and SLE. The optimal extraction conditions were: ethanol:water 90:10 v/v, 50 °C, 5 min, 1:30 *w/v* solid-to-liquid ratio, 135.6 W/cm^2^ ultrasound intensity, and 60 kHz ultrasound frequency. Additionally, Caldas et al. [41] compared conventional (SLE) and non-conventional extraction techniques (UAE and MAE) for the recovery of phenolic compounds from grape skins of sparkling production. As a result, UAE had a major recovery (almost 80 mg GAE g^−1^) at the optimized extraction conditions of ethanol:water 50:50 *v/v*, 1:10 *w/v* solid-to-liquid ratio, and 9 min of extraction time.

To select the extraction technique, several issues must be considered. In addition to extraction yield, capital and operating costs, environmental concerns, operating time, or industrial scaling-up must also be considered. It is well known that conventional extraction is easy to implement in industry, its main drawbacks being solvent consumption and extraction time. In this sense, modern technologies are much more attractive, since solvent consumption is reduced to a significant degree, extraction time is also reduced, and they provide higher yields. In certain instances, the combination of more than one technique can also be considered, either sequentially or hyphenated, such as UAE-SFE, UAE-MAE, EAE-PLE, or PEF-UAE. However, there are scarce applications of modern extraction techniques at an industrial scale, even at a pilot scale [104]. Scaling up from laboratory to industrial scale is not a trivial issue. Hence, the most mature techniques would be UAE or SFE.

### 5.2. Conditioning of Polyphenol Extracts: From Particulate Matter and Colloidal Removal to Volume Size Reduction and Concentration

After the extraction process, extracts of great complexity are obtained, so it is necessary to subject these extracts to a purification stage. This can be accomplished by membrane separation technology, adsorption-desorption, or a combination of both, which leads to obtaining a final product rich in polyphenols, with a higher quality, which can be subsequently used in the food, cosmetic and pharmaceutical industries.

Membrane separation processes can be used at large scale, with three main objectives: i) to remove suspended and colloidal matter in the extraction stage by using pressure-driven membrane techniques as microfiltration (MF 0.1–5 μm, 1–10 bar) and/or ultrafiltration (UF 0.5–100 nm, 1–10 bar); ii) to selectively separate polyphenols from other families of compounds co-extracted in the extraction (e.g., sugars, proteins, lipids) by using pressure-driven membrane techniques as ultrafiltration (UF) and/or nanofiltration (NF) and; iii) to reduce the volume of the polyphenols rich streams by using pressure-driven membrane techniques as nanofiltration (NF 5–10 nm, 10–30 bar) and/or reverse osmosis (RO < 0.5 nm, 35–100 bar) [20,105,106,107,108,109,110,111,112,113].

**Figure 8:**
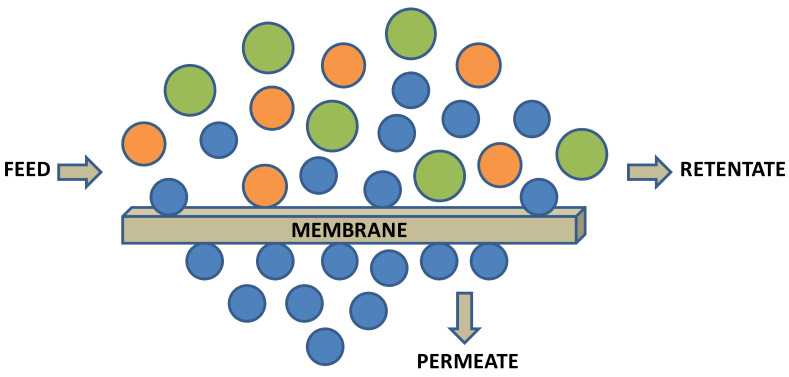
Membrane separation scheme.

Membranes are selective barriers that allow compounds to pass particulate matter (e.g., MF, UF), or dissolved solutes according to their molecular weight cut-off (MWCO) (e.g., UF) or according to the solute properties and active layer (e.g., NF and RO) [110]. Membrane operations separate a solution (feed) into two fractions that are the permeate or filtrate and the retentate or concentrate. The permeated liquid consists of the solvent and solutes that pass through the MWCO of the membrane. The retentate or concentrate contains particles and dissolved compounds partially retained by the membrane, as represented in Figure 8 [4,6].

The separation efficiency of these methodologies depends on a series of factors: membrane characteristics (material, configuration of the separation module, pore size); physical-chemical composition of the solution (type, weight, polarity, solute load), and operating parameters (feed flow rate, trans-membrane pressure, temperature, permeate flow), among others [6].

Membrane technologies’ advantages are low-temperature operation and energy consumption, high separation efficiency, simple equipment, easy scale-up, simple operation, high productivity in terms of permeate flows, and absence of phase transition [4,6]. Compared with traditional concentration methods (evaporation, spray drying, etc.), by membrane processes, the product is not subjected to high temperatures and there is no change in the physical state of the solvent, which means that the functional properties of the compounds of interest are preserved [105].

The integration of membrane processes allows the recovery of purified polyphenol streams and high-quality water for reuse purposes. MF is used to remove suspended solids and prevent the early fouling of membranes in the posterior steps. UF membranes separate the polyphenols in the permeate stream from the macromolecules that remain in the retentate (e.g., sugars, lipids, proteins). NF is adequate to selectively recover and concentrate low-molecular-weight polyphenols. Finally, RO is used for purification purposes [6].

Membrane separation processes have shown to be a suitable stage of pre-treatment for polyphenol recovery in olive mill [20,44,49,51,106,114,115,116,117,118,119,120,121] and winery [36,61,108,110,122,123,124,125] residues. However, more studies are needed for the optimization and implementation of these technologies at an industrial level, for solid and liquid wastes. Examples of the application of different membrane techniques for the recovery of polyphenols from the olive mill and winery wastes are presented in Table 4.

For instance, Zagklis et al. [113] proposed a design of a purification plant for the recovery of polyphenols from olive mill wastewater, grape marc, and olive leaves. The proposed process was based on an integration of UF (tubular ceramic zirconia membrane), NF (spiral wound polymeric membrane), and RO (spiral wound polymeric membrane) techniques, and a final adsorption step. As a result, they obtained 378, 98, and 190 g GAE L^−1^ for olive mill wastewater, olive leaf extract, and grape marc extract, respectively. Additionally, Giacobbo et al. [122], evaluated MF for the recovery of polyphenols from second racking wine lees, with or without previous water addition to the effluent. MF was in total recirculation mode (V0.2 and MFP5, flat-sheet membranes) and in concentration mode (Plasma membrane-associated membranes (PAM) hollow fiber). After MF experiments, a sequential design of UF (ETNA01PP and ETNA10PP membranes) and NF (NF270 membrane) was employed in order to fractionate polyphenols and polysaccharides from wine lees [123]. MF led to obtaining a limpid permeate rich in polyphenols. UF has proven to be effective to separate the polysaccharides of the polyphenols, and NF membrane presented polyphenols rejections higher than 92%, achieving concentrated solutions with high antioxidant activity.

### 5.3. Recovery of Polyphenols by Adsorption Technologies

Due to their low toxicity, chemical stability, selectivity, high adsorption capacity, and ease of regeneration, resins are an attractive option for the purification of phenolic compounds [127,128,129].

Resins are synthetic polymeric adsorbents that include materials such as polystyrene-divinylbenzene copolymers, polymethacrylate, etc., and can be functionalized with ion-exchange groups [128]. The adsorption properties of resins are related to a polymer composition, surface properties, pore structure, and solubility of adsorbents [130].

Adsorption on resins, and further desorption, allows recovering, purifying, and concentrating target compounds simultaneously, to obtain a final product rich in phenolic compounds. The adsorption operation consists of the interaction between the adsorbent (e.g., the resin) and the molecules in the liquid phase, by Van der Waals forces, hydrogen bonds, ionic interactions, or hydrophobic interactions. Adsorption should be reversible when it comes to recovering solute [127].

Adsorption isotherms inform about the adsorption capacity of the resin and how it interacts with the adsorbent. The isotherm provides a relationship between the concentration of the phenolic compounds in solution and the concentration of phenolic compounds adsorbed on the solid phase when both phases are in equilibrium. Langmuir, Freundlich, Temkin, BET, Redlich-Peterson or Dubinin–Radushkevich isotherm models are used to fit the data [131].

For polyphenol recovery, both non-functionalized and ion exchange resins have been proposed—non-ionic resins being the most applied [132]. Pinelli et al. [133] reported that, in general, non-ionic resins show a better performance than ion-exchange resins, but this mode can be advantageous for some specific applications [134].

The nature of the solvent has a strong influence on adsorption. Water or water containing a low percentage of an organic solvent, such as ethanol, are well suited for the loading step. pH may also have a relevant impact on polyphenol adsorption/desorption since it determines polyphenols speciation; at low pH, phenolic compounds are undissociated, while at higher pH values dissociation of carboxylic and phenolic groups can occur. Consequently, the mechanisms of interaction of the resin-polyphenol system are pH-dependent. Ionic strength may also affect the interactions, especially in the ion-exchange mode [128].

Regarding the desorption step, it is important to consider the solubility of the target compounds, but also the compliance with regulations applying to the potential application sectors: food additives, nutraceuticals, etc. Thus, most applications use ethanol or ethanol-water mixtures with a high percentage of ethanol as desorption reagents.

A large variety of resins, such as amberlite XAD 4 [52,115], XAD 16N [115], XAD7HP [52,115], XAD 16 [52,133,135], IRA-958 Cl [133], IRA-67 [133,134], FPX-66 [52], Lewatit AF5, AF6, AF7, MonoPlus M800, K6387, VP OC 1600, MonoPlus SP112, CAL I [52], Macronet MN-202 [114], Amberlyst A26 [134], have been reported for the recovery of polyphenols in olive mill wastewater.

For instance, Savarese et al. [114] proposed a combination of membrane tangential filtration (UF, NF, and RO) and adsorption techniques at a pilot scale for the treatment of wastewater from oil mills and the recovery of a polyphenol concentrate stream. For membrane filtration, polysulfone spiral-wound polymeric membranes were used, with an antifouling treatment. The NF concentrate was sent to the section of adsorption on a resin, for concentration and purification. A non-ionic, highly cross-linked, polymeric adsorbent (polystyrene–divinylbenzene) was used (Macronet MN-202), characterized by a high specific surface area (800–1100 m^2^ g^−1^). The phenolic compounds retained on the column during the adsorption phase were desorbed with ethanol. As a result, they produced 62% purified water which could be reused in industrial processes, and a concentrated phenolic extract (9.5% of total phenols), with the capacity to be used in the food, cosmetic or pharmaceutical industries.

Soto et al. [136] investigated the adsorption of phenolic compounds from white wine vinasses, onto non-ionic resins, with the objective of recovering phenolic compounds from the effluent. The adsorption process in the column was evaluated by modifying the system variables (polyphenols concentration, flux, and bed height). PS-DVB copolymers with different hydrophobicity, Sepabeads SP700, and Amberlite XAD16HP were used. The increase in bed depth from 10 to 20 cm, increased the breaking point time (t_b_) from 19.6 to 25.6 min, resulting in higher removal efficiency of phenolic compounds. The maximum removal capacity (q_Th_) calculated by the Thomas model reached 98.3 mg g^−1^ at a flow rate of 5 mL min^−1^.

Frascari et al. [132] developed and applied a procedure for the selection of the optimal adsorbent for phenolic compounds recovery from olive mill wastewaters. They compared four non-functionalized resins (Amberlite XAD16N, Optipore SD-2, Amberlite FPX66, Amberlite XAD761) and one ion-exchange resin (Amberlite IRA958 Cl). In the initial batch isotherm tests, best results were obtained by neutral resin XAD16N and the ion-exchange resin IRA958 (used in OH^-^ form), with phenolic sorption capacities of 81 and 48 mg g^−1^ dry resin, respectively; and phenolic content in the desorbed product of 0.19 and 0.21 g g^−1^ volatile solids, respectively. In continuous-flow adsorption/desorption tests, results indicated that at low (20%) breakpoint, XAD16N had a better performance (0.14 g g^−1^ volatile solids), but at high (90%) breakpoint the two resins produce similar results in terms of both phenolic content (0.19–0.21 g g^−1^ volatile solids) and antioxidant capacity (4.6–4.9 g ascorbic acid equivalent g^−1^).

Kaleh and Geißen [52] isolated valuable biophenols from olive mill wastewater, using the procedures of acidification, sedimentation, and membrane filtration (MF and UF). Adsorption of hydroxytyrosol, tyrosol, caffeic acid, oleuropein, and luteolin was investigated onto 16 commercial sorbents (Amberlite XAD-4, XAD-7HP, XAD-761, XAD-16 nonionic, FPX-66, PVPP, Lewatit AF5, AF6, AF7, MonoPlus-M800, VP OC-1064-MDPH, VP OC-1600, MonoPlus-SP 112, K6387, CAL I and GAC) in a multi-compound model and natural olive mill wastewater. MIPs were also tested. AFs resins demonstrated the highest selectivity for hydroxytyrosol and tyrosol while the desorbed ratios were much higher for the MIPs [52].

In this context, the studies carried out to date show that the purification of polyphenol extracts by using polymeric resins is an interesting alternative, especially when applied after a previous separation/concentration stage by filtration through membranes.

## 6. Conclusions

The growing number of wastes from the olive oil and wine industries lead to the necessity to reuse these waste materials and develop further processing technologies, following circular economy schemes, for their beneficial application. Furthermore, the high costs of waste disposal make necessary its recovery, especially for these industries, which use large-scale production processes. Thus, the recovery of polyphenols that are high added-value compounds from these wastes is both industrially sustainable and environmentally friendly. For that, there exist innovative methodologies to analyze, extract, separate, and purify these bioactive compounds with promising results. However, more studies are needed to improve these processes and to expand their application to a wider set of polyphenols with the necessary quality to be used in the food, cosmetic and pharmaceutical industries.

## Figures and Tables

**Table 1:** Phenolic compounds in olive mill wastes.

Sample	Identified Phenolic Compounds	Concentration	Reference
Olive pomaces	Hydroxytyrosol	5.3–512.6 mg kg^−1^ dw	[14,15]
	Tyrosol	886.7 mg kg^−1^ dw	
	Oleuropein	<0.5–162.9 mg kg^−1^ dw	
	3,4-dihydroxybenzoic acid	37.2 mg kg^−1^ dw	
	Vanillic acid	21.6 mg kg^−1^ dw	
	Homovanillic acid	12.5 mg kg^−1^ dw	
	*p*-hydroxybenzoic acid	3.3 mg kg^−1^ dw	
	Luteolin	32.9–410.9 mg kg^−1^ dw	
	Rutin	1.3–354.2 mg kg^−1^ dw	
	Caffeic acid	0.7–876.2 mg kg^−1^ dw	
	Chlorogenic acid	9.7–47.7 mg kg^−1^ dw	
	Ferulic acid	6.1–34.6 mg kg^−1^ dw	
	*p*-coumaric acid	8.0–67.1 mg kg^−1^ dw	
	Quercetin	0.5–36.6 mg kg^−1^ dw	
	Naringenin	0.9–3.3 mg kg^−1^ dw	
Olive mill wastewaters	Hydroxytyrosol	102–1409 mg L^−1^	[16]
	Tyrosol	14–425 mg L^−1^	
	Caffeic acid	1–4 mg L^−1^	
	Elenolic acid	87–1884 mg L^−1^	
	Salidroside	33–265 mg L^−1^	
	Comselogoside	1–2 mg L^−1^	
	Hydroxytyrosol 4-O-Glucoside	54–3150 mg L^−1^	
	Hydroxytyrosol 1-O-Glucoside	23–27 mg L^−1^	
	Hydroxytyrosol Glycol	132–325 mg L^−1^	
	Ester of caffeic	1 mg L^−1^	

dw, dry weight.

**Table 2:** Phenolic compounds in winery wastes.

Sample	Identified Phenolic Compounds	Concentration	Reference
Grape pomaces	*p*-hydroxybenzoic acid	6.03–50.9 mg kg^−1^ dw	[28]
	Protocatechuic acid	4.34–57.4 mg kg^−1^ dw	
	Gallic acid	149–987 mg kg^−1^ dw	
	Ellagic acid	196–1040 mg kg^−1^ dw	
	Vanillic acid	97.5–302 mg kg^−1^ dw	
	Syringic acid	24.1–660 mg kg^−1^ dw	
	*p*-coumaric acid	2.85–77.4 mg kg^−1^ dw	
	Chlorogenic acid	4.51–102 mg kg^−1^ dw	
	Caffeic acid	75.1–82.8 mg kg^−1^ dw	
	Resveratrol	6.10–78.0 mg kg^−1^ dw	
	Quercetin	547–848 mg kg^−1^ dw	
	Rutin	8.11–569 mg kg^−1^ dw	
	Kaemferol	454–553 mg kg^−1^ dw	
	Catechin	403–3711 mg kg^−1^ dw	
Grape stems	Gallic acid	70.4–469 mg kg^−1^ dw	[29]
	(+)-Catechin	385–1858 mg kg^−1^ dw	
	(+)-Epicatechin	12.3–189 mg kg^−1^ dw	
	Procyanidin B3	138–993 mg kg^−1^ dw	
	Procyanidin B2	36.0–165 mg kg^−1^ dw	
	Epicatechin gallate	34.2–130 mg kg^−1^ dw	
	trans-Caftaric acid	5.1–274 mg kg^−1^ dw	
	trans-Resveratrol	74.0–266 mg kg^−1^ dw	
	3-Viniferin	167–499 mg kg^−1^ dw	
Wine lees	(+)-Catechin	43.1–50.1 mg L^−1^	[30]
	(-)-Epicatechin	7.7–517.1 mg L^−1^	
	Procyanidin B1	15.3–46.8 mg L^−1^	
	Procyanidin B2	19.4–29.7 mg L^−1^	
	Myricetin	1.3–1.8 mg L^−1^	
	Quercetin	4.2 mg L^−1^	
	Gallic acid	8.1–35.9 mg L^−1^	
	trans-caftaric acid	21.2–23.3 mg L^−1^	
	trans-coutaric acid	1.3–9.6 mg L^−1^	
	Caffeic acid	0.7 mg L^−1^	
	*p*-coumaric acid	0.6–0.9 mg L^−1^	
	Ferulic acid	0.2–0.9 mg L^−1^	

dw, dry weight.

**Table 3:** Extraction techniques for polyphenols recovery from the olive mill and winery wastes.

Technique	Sample	Solvent	Experimental Conditions	Polyphenols Concentration	Reference
Olive Mill Residues					
Liquid–liquid extraction	Olive mill wastewater	Ethyl acetate	25 °C, four extraction cycles	6.490 ± 0.063 g GAE L^−1^	[50]
Liquid–liquid extraction	Olive mill wastewater	Ethyl acetate	25 °C, 20 min, four extraction cycles	1407 mg GAE L^−1^	[89]
Liquid–liquid extraction	Olive mill wastewater	Ethyl acetate	1:1 *v/v*, 25 °C, two extraction cycles	8.90 ± 0.728 mg GAE L^−1^	[90]
Liquid–liquid extraction	Olive mill wastewater	Ethyl acetate	25 °C, three extraction cycles	9.8 g tyrosol equivalents L^−1^	[21]
Liquid–liquid extraction	Olive mill wastewater	Ethyl acetate	1:2 *v/v*, 27 °C, 30 min	3440 mg GAE L^−1^	[91]
SLE	Olive pomace	Methanol	1:25 *w/v*, 70 °C, 12 h	4.37 mg GAE g^−1^	[45]
SLE	Olive pomace	Ethanol	1:5 *w/v*, 25 °C, 180 min, pH 2	1.23 ± 0.21 caffeic acid equivalents (CAE)	[92]
SLE	Olive pomace	Methanol	1:10, *w/v*, 180 °C, 90 min	45.2 mg CAE g^−1^	[93]
SLE	Dry olive mill residue	Water	1:15 *w/v*, 25 °C, 40 min	25 mg GAE g^−1^	[94]
SLE	Olive pomace	Dimethyl sulfoxide	1:3 *w/v*, 25 °C, 30 min	1.3 g kg^−1^	[48]
SLE	Olive pomace	Ethanol:water 80:20 *v/v*	1:2 *w/v*, 25 °C, 120 min	171 ± 4 mg of gallic acid 100 g^−1^	[95]
SLE	Olive leaves	Dimethyl sulfoxide	1:15 *w/v*, 25 °C, 30 min	50 g kg^−1^	[48]
MAE	Olive leaves	Water	86 °C, 3 min	104.22 ± 0.61 mg GAE g^−1^	[96]
UAE	Olive pomace	Isopropanol:water 1:1 *v/v*	1:5 *w/v*, 25 °C, 40 min	69.66 mg GAE g^−1^	[47]
UAE	Olive pomace	Ethanol:water 90:10, *v/v*	1:30, *w/v*, 50 °C, 5 min	55.1 mg g^−1^ hydroxytyrosol, 381.2 mg g^−1^ maslinic acid and 29.8 mg g^−1^ oleanolic acid	[97]
UAE	Olive pomace	Water	1:50 *w/v*, 30 °C, 75 min	19.71 ± 1.41 mg GAE g^−1^	[98]
PLE	Olive pomace	Ethanol:water 50:50 *v/v*	120 °C, 20 min	5.8% extraction yield (8 gr)	[53]
PLE	Olive leaves	Ethanol:water1234550:50 *v/v*	80 °C, 5 min	53.15 mg GAE g^−1^	[99]
SFE	Olive pomace	Carbon dioxide	40 °C, 350 bar, 60 min	0.76 ± 0.15 CAE	[92]
PEF	Olive leaves	Ethanol:water (25% EtOH)	Pulse duration: 10 µs, pulse period: 1000 µs, electric field 1 kV cm^−1^, time: 30 min	20.75 mg GAE g^−1^	[86]
Winery residues					
SLE	Grape pomace	Ethanol:water 50:50 *v/v*	1:25 *w/v*, 60 °C, 2 h	196.2 ± 22.7 mg GAE g^−1^	[58]
SLE	Grape pomace	Ethyl acetate	1:10 *w/v*, 25 °C, 6 h	70.5 ± 0.03 mg GAE g^−1^	[28]
SLE	Grape skins	Ethanol	0.10:1 *w/v*, 25 °C, 19 h	3.22 mg GAE g^−1^	[55]
SLE	Grape pomace	Acetone	1:12.5 *w/v*, 60 °C, 45 min	31.25 mg GAE g^−1^	[100]
SLE	Wine lees	Methanol/2% HCl 95:5 *v/v*	1:5 *w/v*, 25 °C, 60 min, three extraction cycles	2316.6 ± 37.9 mg GAE 100 g^−1^	[60]
UAE	Grape skins	Ethanol:water 50:50 *v/v*	1:10 *w/v*, 28 °C, 9 min	80 mg GAE g^−1^	[56]
UAE combined with SFE	Grape pomace	UAE: Ethanol:water (ethanol concentration 449.73 g L^−1^), SFE: Carbon dioxide	UAE: 1:4 *w/v,* 80 °C, 4 min SFE: 8 MPa, 40 °C, 30 min	3493 mg GAE 100 g^−1^	[101]
UAE	Grape pomace	Ethanol:water 1:1 *v/v*	1:70 *w/v*, 20 °C, 60 min	438984 ± 4034 ppm GAE	[57]
UAE	Grape seeds, pomace, and stems	Ethanol:water (44% of ethanol)	1:4 *w/v*, ˂ 50°C, 3 min, two extraction cycles	188, 89.15, 63.46 mg GAE g^−1^ for grape seeds, pomace, and stems, respectively	[70]
UAE	Wine lees	Ethanol 43.9%	1:60 *w/v*, 60 °C, 25 min	58.76 mg GAE g^−1^	[102]
PLE	Grape pomace	Ethanol:water 50:50 *v/v*	80 °C, 50 min, 100 bar	79 g GAE kg^−1^	[103]
EAE	Grape seeds	Water, pH 3.55, Lallzyme EX-V	Enzyme dosage 20 mg g^−1^, 48 °C, 2.60 h	Flavan-3-ols 21.41 ± 21 mg kg^−1^ Gallic acid 227.04 ± 0.35 mg kg^−1^	[84]
OH	Grape pomace	Ethanol:water (30% ethanol)	400 V cm^−1^, 50 °C, 60 min	620 mg GAE 100 g^−1^	[85]

**Table 4:** Membrane techniques for polyphenols recovery from the olive mill and winery wastes.

Technique	Sample	Membrane	Polyphenols Concentration	Reference
Olive Mill Residues				
MF, UF and NF	Olive mill wastewater	Permapore EOV 1046 (MF), Permapore DGU 1812 BS EM (UF) and PERMAPORE AEN 1812 BS (NF)	2456 to 5284 μg mL^−1^ (MF) 1404 to 3065 μg mL^−1^ (UF) 373 to 1583 μg mL^−1^ (NF)	[44]
MF and UF	Olive mill wastewater	Microlab 130 S (MF) and 18 PCI (UF)	7.2 g L^−1^ of hydroxytyrosol	[117]
MF, UF and NF	Olive mill wastewater	Becopad P550 (MF) and PES spiral membrane from 100 kDa and 3–5 Da MWCO	250, 250 and 430 mg in the NF retentate of the first, second, and third treatment, respectively	[118]
NF and RO	Olive pomace	NF270 (NF), NF90 (NF) and BW30 (RO)	1063.9, 1069.4 and 1234.3 mg GAE L^−1^ for NF270, NF90 and BW30, respectively	[106]
MF, UF, NF and RO	Green leaves, dried leaves, and pitted olive pulp	Tubular ceramic membranes in titanium oxide (MF) and spiral wound module membranes in PES (UF, NF and RO)	244.15, 57.63 and 289.93 mg g^−1^ for green leaves, dried leaves, and pitted olive pulp, respectively	[126]
UF, NF and RO	Olive mill wastewater, grape marc, and olive leaves	Tubular ceramic zirconia membrane (UF) and spiral wound polymeric membrane (NF and RO)	378, 98, and 190 g GAE L^−1^ for olive mill wastewater, olive leaf extract and grape marc extract, respectively	[113]
MF and RO	Olive mill wastewater	Membralox EP19-40 (MF) and SW30HR (RO)	1070 mg L^−1^	[109]
Winery residues				
MF, UF and NF	Vinasses	Iberlact (MF and NF) and Tami (UF) membranes	0.45 g GAE g^−1^	[61]
MF	Wine lees	V0.2 and MFP5, flat-sheet membranes, and plasma membrane-associated membranes (PAM) hollow fiber	26.1 mg GAE L^−1^	[122]
MF	Wine lees	PVDF flat-sheet membrane with 0.2 mm pore size and polyimide hollow fiber membrane with 0.4 mm pore size	1 g GAE L^−1^	[108]
UF and NF	Wine lees	M-U2540 (UF), ESP04 (UF), HYDRACoRe 70pHT (NF), NF270 (NF), NF90 (NF), and HFW1000 (NF)	2.65 g GAE L^−1^	[124]
MF and NF	Wine lees	Polyvinylidenefluoride (PVDF) hollow fiber membranes (MF), NP010 (NF), NP030 (NF) and MPF36 (NF)	982.1 mg GAE L^−1^	[107]

## Data Availability

Data presented in this study is available on request from the corresponding authors.
